# Formula with large, milk phospholipid-coated lipid droplets in late-moderate preterm infants: a double-blind RCT

**DOI:** 10.1038/s41390-024-03476-x

**Published:** 2024-09-18

**Authors:** Andreas Kakaroukas, Marieke Abrahamse-Berkeveld, Louise Hayes, Richard J. Q. McNally, Janet E. Berrington, Ruurd M. van Elburg, Nicholas D. Embleton

**Affiliations:** 1 Newcastle Neonatal Service, Royal Victoria Infirmary, Newcastle upon Tyne Hospitals National Health Service (NHS) Foundation Trust, Newcastle upon Tyne, UK; 2 Emma Children’s Hospital, Amsterdam University Medical Centers, Amsterdam, The Netherlands; 3 Danone Nutricia Research, Utrecht, The Netherlands; 4 Faculty of Medical Sciences, Population Health Sciences Institute, Newcastle University, Newcastle upon Tyne, UK; 5 Translational and Clinical Research Institute, Newcastle University, Newcastle upon Tyne, UK

## Abstract

**Background:**

Limited evidence exists on the preferred feeding method when breastfeeding is not possible in late and moderate preterm (LMPT) infants. This RCT evaluates growth, safety, and tolerance of a concept infant formula (IF) with large, milk phospholipid-coated lipid droplets enriched in dairy lipids in LMPT infants with primary objective to demonstrate non-inferiority of daily weight gain from randomization to 3 months corrected age compared to a standard IF.

**Methods:**

LMPT infants were randomized before or around term equivalent age to either the concept (*n* = 21) or standard IF (*n* = 20). Forty-one breastfed (BF) infants served as reference.

**Results:**

Due to unintended low recruitment, non-inferiority in daily weight gain could not be demonstrated for the Concept compared to the Control group, but was compared to the BF group. Other outcomes were similar between the formula groups, except for an apparent larger head circumference gain in the Concept group. No apparent differences in growth and body composition outcomes were observed between the Concept and BF reference groups.

**Conclusion:**

This small-scale study suggests the concept IF is a safe alternative for parents who choose IF to feed their LMPT infant. Larger trials are needed to better determine impacts on head growth or body composition.

**Impact:**

- In a small group of late and moderate preterm infants, growth from randomization until 3 months corrected age of infants fed with a concept infant formula with large, milk phospholipid-coated lipid droplets was not -significantly different from infants fed a standard infant formula.
- Infants in the Concept group had non-significant larger gain in head circumference compared to the Control group; larger trials are needed to confirm this finding.
- Both formulas were well-tolerated, with no differences in adverse events.
- The concept formula is potentially a safe alternative for parents of moderate to late preterm infants who choose to use formula milk.

## Introduction

Late and moderate preterm (LMPT) infants are at risk of short- and long-term adverse metabolic and cognitive outcomes and may also experience growth faltering in infancy^1–3^. Studies in LMPT infants show a greater risk for cardiometabolic disease in childhood and adult life compared to term infants^4–7^. LMPT infants are also more likely to develop neurodevelopmental delay and cerebral palsy^8–13^.

Nutrition, and especially exposure to breast milk, plays an important role in modulating these risks. In LMPT infants breastfeeding has a beneficial impact on body composition, cognitive and metabolic outcomes^14–18^. Providing support to mothers to breastfeed is vital^5,19,20^. Partial or full supplementation of feeds with an infant formula (IF) is often required, especially in preterm neonates. Given the inconsistency in practice, research is needed to provide evidence for the type of IF supplementation for this population.

Lipids are a vital dietary component providing energy and essential fatty acids and enabling transport and uptake of fat-soluble vitamins. Lipids in breast milk have unique characteristics and predominantly consist of triglycerides^21^. The lipid droplets are large (0.1–15 μm diameter with average diameter of ~4 μm) and have triglycerides in the core covered by a three-layered native membrane primarily consisting of phospholipids, sphingomyelin, glycolipids, cholesterol, and functional proteins^22,23^. The lipid droplets in IFs are smaller (mode diameter 0.4 μm) and proteins are the main emulsifiers^24^. A concept IF has been developed with larger lipid droplets that provide triglycerides in the core coated by an interface of phospholipids, sphingomyelin, glycolipids and proteins, and cholesterol, which may better mimic the properties of human breast milk^24^. This concept IF has been shown to support adequate growth in term infants in the first 4 months, with equivalence in daily weight gain compared to infants fed standard IF ^25,26^.

We established a cohort of LMPT infants to study growth and body composition, feeding and eating behavior, and neurodevelopmental outcomes in the first 2 years and recently reported growth and body composition outcomes at 3 months corrected age^18^. Within this cohort we nested a non-inferiority, double-blind randomized control trial (RCT) that compared outcomes on infants fed the concept IF versus a standard IF. The primary outcome was daily weight gain between randomization to 3 months corrected age. Secondary outcomes until 3 months corrected age included growth and body composition parameters from term equivalent age (TEA)(40^+0^ weeks corrected gestation) until 3 months corrected age (3 months after TEA), gastrointestinal tolerance as well as safety outcomes.

## Materials and methods

The study was reviewed by the North East–York Research Ethics Committee and approved by the NHS Health Research Authority (IRAS project ID: 237542, 11/04/2018) and registered at ISRCTN (ISRCTN15469594). Consent was obtained from the parents according to the Good Clinical Practice.

### Participating centers

The study was conducted at the Royal Victoria Infirmary (RVI), Newcastle upon Tyne, in compliance with Baby Friendly Initiative recommendations. Exclusively formula fed infants who were born in four other regional hospitals and lived within traveling distance from Newcastle were also eligible for the trial.

### Participants

Infants were screened for eligibility for the trial. Inclusion criteria were gestational age between 32 + 0 and 36 + 6 weeks, birth weight between 1.25 and 3.0 kg, age less than 4 weeks after TEA (4 weeks corrected age), prior enrollment to the over-arching cohort study (see below), and exclusive formula feeding (the initial criteria of a birth weight between 1.25 and 2.5 kg and randomization prior to TEA were adapted 9 months after the study started due to low recruitment). Infants who had known significant health problems that could impact their growth or with child protection or other social concerns that could affect the follow-up were excluded.

### Measurements

The design and protocol of the cohort have previously been published^27^, and we summarize here the design of the RCT until 3 months corrected age.

Infants born at LMPT gestational age regardless of the type of milk they received were approached to join the cohort study; the potential of joining the RCT was not offered at the initial consent stage to the cohort, in order not to adversely impact breastfeeding. Subsequently, only infants who were exclusively formula-fed before 4 weeks corrected age were approached for a second consent to take part in the RCT.

Enrolled infants were randomized to receive either a concept IF or a standard IF using an online platform (www.sealedenvelope.com). The two formulas used in the RCT were coded with the letters A, B, C, and D (two letters for each type of milk). Both parents and the research team remained blinded to the type of milk that the infants received throughout the study. Parents were asked to introduce the study formula within 3 days of enrollment in the RCT. The intervention was completed at 6 months corrected age. Siblings of multiple births were assigned to the same intervention group. Breastfed infants participating in the cohort were considered as the reference group.

Data were collected at specific time points; at enrollment to the cohort and/or randomization to the RCT, at TEA, and at 3 months corrected age. During these visits, anthropometric measurements were performed, including weight, length, head circumference, mid-upper arm and thigh circumferences, skinfolds (triceps, biceps, sub-scapular, and supra-iliac). Measurements were performed by the same two investigators, according to the WHO methodology for anthropometric measurements. These were standardized to z-scores for the corrected age where available, using the WHO anthro software^28^ (for measurements after corrected term gestation) and the LMS growth data (for ages before TEA)^29^. Dual X-Ray absorptiometry was performed at, or before TEA and at 3 months corrected age to assess body composition. During the visits, questionnaires were completed by parents on the infant’s general health, history of medication and/or supplementation, feeding behavior, intake and tolerance.

Secondary outcomes presented here include gastrointestinal symptoms and milk tolerance, stool frequency, and consistency. Infants in the RCT completed a 7-day diary, prior to each visit. Parents recorded the number of feeds, the volume of formula consumed, the stool frequency and type/consistency. The diary was also used to record the frequency and intensity of symptoms including vomiting, regurgitation, colic, and nappy rash. Parents also completed the infant gastrointestinal symptoms questionnaire (IGSQ) and the Baby Eating Behavior Questionnaire (BEBQ), which are validated tools to assess symptoms of feeding tolerance and feeding behavior respectively, based on the parental perspective ^30^.

### Study products

Both intervention formulas were produced by Danone Nutricia Research according to the good manufacturing practices (ISO 22000) and the Directive 2006/141/EC on composition of IFs. They had similar appearance and taste. Concentration in calories (66 kcal/100 ml) and nutrients was highly similar (protein 1.3 g/100 ml, lipids 3.4–3.5 g/100 ml). Both formulas were cow’s milk-based IF containing intact protein with a casein:whey ratio of 5:8, prebiotics (0.8 g/100 ml): short-chain galacto-oligosaccharides (scGOS) and long-chain fructo-oligosaccharides in a ratio of 9:1 and long-chain polyunsaturated fatty acids (PUFA) Docosahexaenoic Acid (DHA):Arachidonic Acid (AA) in a ratio of 10:11 (derived from fish and algae oil)(Supplementary Table 1). The key differences were the size of the lipid droplets, the coating of the lipid droplets, and the origin of the lipids. The concept IF contained a mixture of vegetable (46%) and dairy (54%) lipids, including milk phospholipids, with more than 3 times more sn-2 palmitic acid compared to the control IF (35% and 10% respectively). The lipid droplets in the concept IF had a mode diameter of 3–5 μm, comprising the triglycerides in the core and an interface predominantly composed of milk phospholipids following an adapted production process (Nuturis®)^24^. The control IF was a standard IF comprised of vegetable oils which were present as lipid droplets with an average size of 0.5 μm and proteins as their main emulsifiers (no milk phospholipids present).

### Data analysis

The primary outcome was daily weight gain between the time of randomization and 3 months corrected age. We hypothesized that the weight gain of infants fed the concept formula would be non-inferior to those fed the control IF. Non-inferiority is established if the lower bound of a calculated two-sided 90% confidence interval (CI) is above the non-inferiority margin. The American Academy of Pediatrics (AAP) guidelines^31^ suggest 3 g/d is a clinically relevant difference of weight gain for the first 3 months in term infants. Therefore, we selected a non-inferiority margin of −3 g/d clinically relevant assuming a standard deviation (SD) of 6 g/d^31^. Based on the above margin, a significance level of 5% and power of 80%, fifty infants would be required in each group. Assuming 30% attrition we aimed to recruit ~140 infants in the trial (allocation ratio in the two groups 1:1).

In line with standard practice for growth adequacy and safety studies, we considered the per protocol analysis as leading for the primary outcome^27^. This approach increases the possibility of detecting a difference in the primary outcome in this non-inferiority study since the compliance to exposure to the intervention formulas was high in this population. There were no infants with any major breach of the protocol.

For continuous outcome parameters with normal distribution the mean values and standard deviation were calculated, and t-test was used for comparison between groups. Median and quartiles 1 and 3 were calculated for values with non-normal distribution and Mann-Whitney test was performed to compare variables between groups. A Pearson chi-square test was used to compare categorical variables.

Significance testing was performed for all comparisons using two-sided testing with a significance level of 5% and a two-sided confidence level of 95% A resultant probability value of *P* < 0.05 was considered statistically significant. Given the exploratory nature of the study, the interpretation of findings was focussed on the effect size of potential differences rather than on the *p* value itself.

## Results

**Fig. 1:**
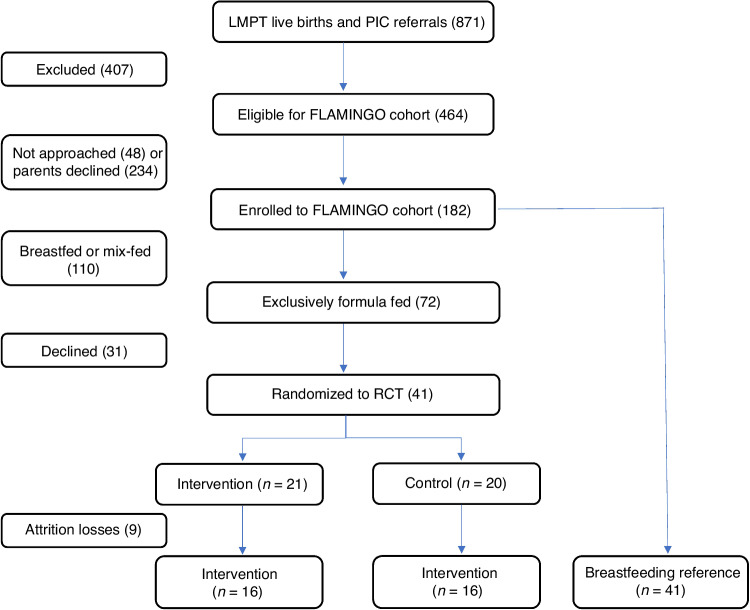
Recruitment process of the FLAMINGO RCT. RCT randomized control trial, LMPT late and moderate preterm, PIC patient identification center, 3mCA 3 months corrected age.

Recruitment started in May 2018 and was completed in June 2020 and was affected by the COVID-19 pandemic, resulting in premature cessation of the study. During this period, a total of 852 potentially eligible LMPT infants were born in the RVI and a further 19 eligible, formula-fed newborns were referred from neighboring hospitals. A total of 182 infants were recruited from this group and formed the cohort. Of these, 41 infants who were exclusively formula fed prior to 4 weeks corrected age and whose parents gave a second consent, participated in the RCT (Fig. 1). This number is substantially lower than the aimed sample size of 140 infants. Forty-one cohort infants were exclusively breastfed at the time of enrollment and formed a reference group.

Exclusively formula-fed infants were randomized to receive control IF (Control group, *n* = 20) or the concept IF (Concept group, *n* = 21). Several apparent differences in demographic characteristics were observed between both randomized groups (Table 1). Infants in the Concept group entered the RCT and started the intervention at nearly 2 weeks lower corrected gestation compared to the Control group (median corrected gestation 38.1 weeks and 39.9 weeks respectively). Additionally, when comparing the Concept group to the Control group there were slightly more boys (57% vs 45%), more births via C-section (62% vs 50%), more admissions in the Neonatal Unit (57% vs 45%) and more infants who received antibiotics in the postnatal period (57% vs 40%). Moreover, median maternal BMI as well as mean maternal age was higher in the Control group compared to the Concept group. The demographic characteristics for the reference group and the full study cohort are summarized in Supplementary Table 2.

**Table 1:** Demographic characteristics of the intervention groups and the breastfed reference group

	Control	Concept	Breastfed	*p* value*	*p* value**
	*n* = 20	*n* = 21	*n* = 39		
Male	9 (45%)	12 (57%)	19 (49%)	0.437	0.533
Female	11 (55%)	9 (43%)	20 (51%)		
Gestation (weeks)^a^	34.82 (1.69)	34.82 (1.35)	35.11 (1.28)	0.992	0.412
Birth weight	2.285 (0.331)	2.193 (0.359)	2.239 (0.387)		
Birth weight-for-age z-score	−0.21 (0.66)	−0.47 (1.03)	−0.54 (0.97)	0.347	0.782
Moderate preterm	8 (40%)	6 (29%)	9 (23%)	0.441	0.639
Late preterm	12 (60%)	15 (71%)	30 (77%)		
Corrected gestation at randomization^b^	39.9 (37.5, 40.6)	38.1 (36.6, 40)	36.4 (35.5, 37.1)	0.154	0.063
Mode of delivery					
Normal vaginal delivery	10 (50%)	8 (38.1%)	16 (41%)	0.682	0.864
Elective C-section	3 (15.0%)	5 (23.8%)	7 (18%)		
Emergency C-section	7 (35.0%)	8 (38.1%)	16 (41%)		
Singleton	12 (60.0%)	13 (61.9%)	33 (84.6%)	0.901	0.047
Multiple	8 (40.0%)	8 (38.1%)	6 (15.4%)		
Admitted to Neonatal Unit	9 (45.0%)	12 (57.1%)	17 (43.6%)	0.437	0.316
Postnatal antibiotics	8 (40.0%)	12 (57.1%)	19 (48.7)	0.272	0.533
Days feeding tube support required	1 (0, 6.5) *n* = 20	4 (0, 10) *n* = 21	5 (0, 12)	0.178	0.018
Days received any breast milk	0 (0, 19) *n* = 20	2, (0, 17) *n* = 21	N/A	0.885	N/A
Maternal age (years)^a^	33.5 (7.4)	31.2 (5.5)	33.1 (4.7)	0.277	0.183
Maternal BMI^b^	28.9 (24.3, 31.6)	25.7 (22.9, 32.7)	24.2 (22, 27.2)	0.583	0.361

*p value between control and concept groups.

**p value between concept and breastfeeding groups.

^a^Values are mean (standard deviation).

^b^Values are median (quartile1, quartile 3).

### Growth outcomes at term and 3 months corrected age

The difference in mean daily weight gain from randomization to 3 months corrected age between the Concept and Control groups was −1.28 g/d (90%CI: −4.80, 2.24; *p* = 0.541), not allowing to confirm non-inferiority. In contrast, non-inferior daily weight gain was observed when comparing the Concept group with the breastfed reference group (difference in means of 0.49 g/d (90%CI: −2.88, 3.86)). No statistically significant difference was observed for the mean daily weight gain between the Concept group (29.12 g/d; SD = 6.46) compared to the Control group (30.4 g/d; SD 5.19 *p* = 0.541, Table 2) or the breastfeeding reference group (28.63 g/d, SD 6.90, *p* = 0.804). The two intervention groups had a similar gain in length-for-age z-score and weight-for-age z-score from randomization to 3 months corrected age. Infants in the Concept formula group had a markedly greater positive gain in head circumference z-score compared to those in the Control group (and BF reference group).

**Table 2:** Growth parameters changes in period between randomization and 3 months corrected age

	Control	Concept	Breastfeeding^c^		
	Mean (SD)	Mean (SD)	Mean (SD)	*p* value* CI 95%*	*p* value** CI 95%**
Daily weight gain (g/d)^a^	30.40 (5.19) *n* = 16	29.12 (6.46) *n* = 16	28.63 (6.90) *n* = 39	0.541 −2.950, 5.511	0.804 −3.547, 4.525
Weight-for-age z-score change^a^	0.35 (0.82) *n* = 16	0.34 (0.71) *n* = 16	0.43 (1.13) (*n* = 39)	0.988 −0.549, 0.557	0.764 −0.703, 0.520
Length-for-age z-score change^b^	0.14 (−0.17, 0.71) *n* = 13	0.32 (−0.28, 0.69) *n* = 14	0.75 (1.15) *n* = 35	0.933	0.229
Head circumference-for-age z-score change^a^	0.38 (0.92) *n* = 14	1.12 (0.95) *n* = 14	0.99 (1.10) *n* = 35	0.343 −1.087, 0.392	0.680 −0.544, 0.807

^*^p value and 95% confidence intervals (C.I.) between control and concept groups.

**p value and 95% confidence intervals (C.I.) between concept and breastfeeding groups.

^a^Results are presented as mean with standard deviation and *p* values calculated using t-test.

^b^Results are presented as median with quartile 1, quartile 3, and *p* values calculated using Mann-Whitney test.

^c^Changes for the breastfeeding group were calculated from the time of enrollment to the cohort until 3 months corrected age.

Anthropometric measurements and z-scores at birth, randomization, and 3 months corrected age are presented in Table 3. The two groups had similar growth parameters at birth and similar changes during the intervention period, except for an increase in head circumference-for-age z-score, from a mean 0.42 [SD 1.29] at randomization to 1.25 [SD 1.06] at 3 months corrected age in the Concept group, compared to a mean of 0.32 [SD 0.68] at randomization and 0.52 [SD 0.86] at 3 months corrected age in the control group. The Concept group had a greater reduction in the mean weight-for-age z-score between birth and randomization (−0.47 [SD 1.03] and −1.12 [SD 1.11], respectively) compared to the Control group (−0.21 [SD 0.66] and -0.47 [SD 0.98], respectively).

**Table 3:** Growth measurements and z-scores of the two intervention, formula-fed groups at birth, randomization, and 3 months corrected age (3mCA)

	Control			Concept		
	Birth	Randomization	3mCA	Birth	Randomization	3mCA
	Mean (SD)	Mean (SD)	Mean (SD)	Mean (SD)	Mean (SD)	Mean (SD)
Corrected gestation or age^a^	34.82 (1.69) *n* = 20	39.9 (37.5, 40.6)^b^ *n* = 20	93 (89.5, 96.5)^b^ *n* = 16	34.82 (1.35) *n* = 21	38.1 (36.6, 40.0)^b^ *n* = 21	91.5 (90.5, 96.0)^b^ *n* = 16
Weight (kg)	2.285 (0.331) *n* = 20	2.971 (0.71) *n* = 20	6.03 (0.77) *n* = 16	2.193 (0.359) *n* = 21	2.54 (0.67) *n* = 21	5.69 (0.95) *n* = 16
Weight-for-age z-score	−0.21 (0.66) *n* = 20	-0.47 (0.98) *n* = 20	-0.21 (0.86) *n* = 16	-0.47 (1.03) *n* = 21	−1.12 (1.11) *n* = 21	−0.88 (1.39) *n* = 16
Head circumference (cm)	31.9 (31.1, 32.14)^b^ *n* = 20	34.5 (1.65) *n* = 18	40.7 (1.3) *n* = 15	31.5 (30.4, 32.7)^b^ *n* = 19	33.5 (2.35) *n* = 21	41.8 (1.3) *n* = 14
Head circumference-for-age z-score	−0.14 (-0.64, 0.16)^b^ *n* = 20	0.32 (0.68) *n* = 18	0.52 (0.86) *n* = 15	-0.46 (-1.40, 0.68)^b^ *n* = 19	0.42 (1.29) *n* = 21	1.25 (1.06) *n* = 14
Length (cm)	44.1 (1.77) *n* = 3	48.8 (3.0) *n* = 17	60.2 (2.4) *n* = 15	45.9 (1.56) *n* = 7	46.5 (3.6) *n* = 21	59.7 (3.1) *n* = 14
Length-for-age z-score	−0.81 (0.72) *n* = 3	-0.43 (0.84) *n* = 17	−0.24 (−1.2, 0.24)^b^ *n* = 15	−0.48 (1.49) *n* = 7	-0.91 (1.31) *n* = 21	−0.34 (−2.1, 0.58)^b^ *n* = 14
BMI (kg/m2)^c^	N/A	12.9 (1.6) *n* = 17	16.5 (1.49) *n* = 15	N/A	11.5 (1.4) *n* = 21	16.0 (1.56) *n* = 14
BMI-for-age z-score	N/A	0.43 (0.83) *n* = 9	-0.14 (0.73) *n* = 15	N/A	-0.58 (0.81) *n* = 7	−0.53 (1.07) *n* = 14
Weight-for-length z-score	N/A	0.28 (0.84) *n* = 9	0.003 (0.72) *n* = 15	N/A	-0.69 (0.48) *n* = 7	−0.25 (1.03) *n* = 14

^a^The age at birth and randomization is presented as corrected gestation in weeks. The age at 3mCA refers to the corrected age in days.

^b^values are median (quartile1, quartile 3).

^c^*BMI* body mass index.

### Body composition at term and 3 months corrected age

A Dual Absorptiometry Xray (DEXA) scan was performed at randomization or TEA and 3 months corrected age to estimate fat and lean mass and their percentages, bone mineral density, and content (Table 4). The median increase of lean mass was 1527 g (IQR 1366-2068) for infants in the Concept group, compared to 1250 g (IQR 1209-1321) for infants in the Control group (*p* = 0.086). The median increase in fat mass did not differ significantly between the 2 groups (1104 g [IQR 873g-1455g] for the Concept group and 1009 g [IQR 947g-1305g] for the Control group, *p* = 0.905). Changes in lean mass percentage were similar in all groups.

**Table 4:** Body composition changes between term equivalent age and 3 months corrected age

	Control	Concept	Breastfed	*p* value* C.I. 95%*	*p* value** C.I. 95%**
DEXA findings	*n* = 9	*n* = 10	*n* = 24		
Lean body mass change (g)^a^	1250 (1209, 1321)	1527 (1366, 2068)	1650 (1451, 2005)	0.086	0.496
Fat mass change (g)^a^	1009 (947, 1305)	1104 (873, 1455)	1221 (954, 1428)	0.905	0.724
Lean mass % change^b^	−11.26 (2.60)	−10.61 (4.03)	−11.52 (3.54)	0.693 −3.965, 2.695	0.518 −1.919, 3.732
Fat mass % change^b^	11.24 (2.61)	10.61 (4.03)	11.52 (3.54)	0.693 −2.696, 3.965	0.518 −3.732, 1.919
Lean mass index change (kg/ m^2^)^b^	0.33 (0.82)	0.68 (0.95)	1.24 (1.38) *n* = 23	0.404 −1.209, 0.511	0.259 −1.528, 0.426
Fat mass index change (kg/ m^2^)^b^	2.38 (0.46)	2.41 (1.04)	2.62 (0.85) *n* = 23	0.930 −0.825, 0.759	0.549 −0.912, 0.494
Bone mineral density change (g/cm^2^)^b^	0.04 (0.03)	0.06 (0.04)	0.03 (0.05)	0.259 −0.054, 0.015	0.136 −0.009, 0.063
Bone mineral content change (g/cm)^b^	47.3 (6.9)	58.9 (18.1)	45.5 (14.9)	0.089 −25.107, 1.974	0.032 1.241, 25.559
Skinfolds based findings	*n* = 15	*n* = 14	*n* = 35		
Triceps change (mm)^b^	2.4 (1.5)	3.1 (1.1)	3.7 (1.5)	0.163 −1.721, 0.305	0.175 −1.479, 0.276
Biceps change (mm)^b^	2.1 (1.8)	1.5 (1.7)	2.5 (1.6)	0.389 −0.778, 1.832	0.089 −1.943, 0.138
Supra-iliac change (mm)^b^	1.0 (1.0)	1.5 (1.2)	1.9 (1.7) *n* = 34	0.197 −1.145, 0.306	0.197 −1.415, 0.306
Sub-scapula change (mm)^b^	1.3 (1.3)	1.5 (1.4)	2.3 (1.8)	0.770 −1.151, 0.862	0.141 −1.889, 0.278
Arm muscle area change (mm^2^)^b^	3.56 (1.41)	2.99 (1.68)	3.92 (1.82)	0.325 −0.604, 1.756	0.106 −2.064, 0.206
Arm fat area change (mm^2^)^b^	2.16 (0.95)	2.42 (0.72)	2.93 (1.02)	0.424 −0.904, 0.391	0.093 −1.118, 0.089
Arm fat% change^a^	5.5 (0.9, 8.2)	5.3 (2.7, 9.9)	8.1 (5.1, 11.3)	0.505	0.267

*p value and 95% confidence intervals (C.I.) for difference between concept and control groups.

**p value and 95% confidence intervals (C.I.) for difference between concept and breastfed reference groups.

^a^Results are presented as median with quartile1, quartile 3, and *p* values calculated using Mann-Whitney test.

^b^Results are presented as mean with standard deviation and p-values calculated using t-test.

The changes in bone mineral density between TEA and 3 months corrected age also did not differ significantly between groups. In fact, the mean increase in bone mineral density for the Concept group was 0.06 g/cm^2^ (SD 0.04), compared to the mean of 0.04 g/cm^2^ (SD 0.03) for the Control group (*p* = 0.259), and a mean of 0.03 g/cm^2^ (SD 0.05) for the breastfed group (*p* = 0.136). The mean increase in bone mineral content did seem slightly higher for the Concept group, compared to the Control group (58.9 [SD 18.1] versus 47.3 [SD 6.9] respectively, *p* = 0.089) and the breastfed group (mean 45.5 [SD 14.9], *p* = 0.032).

No apparent differences in skinfold thickness were observed (Table 4).

All growth and body composition parameters at TEA and 3 months corrected age for each group are presented in Supplementary Table 3.

### Feeding tolerance and gastrointestinal symptoms

Stool and tolerance characteristics at 3 months corrected age did not differ between the 2 intervention groups, except for a tendency towards a higher occurrence of regurgitation in the Control group (Supplementary Table 4). Infants in the Control group had more reported days with regurgitation (control mean 58%, SD 45.5, Concept group mean 18.1%, SD 34.7, *p* = 0.053) but similar proportion of days with vomiting (median 13%, IQR 0-100%, Concept group: median 11%, IQR 0-29%, *p* = 0.47) with the Concept group.

The median scores of the IGSQ were similar among the groups. At TEA the median IGSQ was 24 (IQR 20–37) for the Concept group, 25 (IQR 21–29) for the Control group and 25 (IQR 21–31) for the breastfed reference group. At 3 months corrected age median IGSQ score was 22 (IQR 18–25) for the Concept group compared to a median of 20 (IQR 20–28) for the Control group (*p* = 0.74). The median score for the breastfed reference group was 22 (IQR 20–26). No differences in formula intake were observed between both intervention groups (Supplementary Table 4). The two formula groups had similar mean scores for the BEBQ as well at TEA (data not shown) and 3 months corrected age (Concept 50.3, SD 6.9 and 47.9, SD 7.0 respectively; Control 50.5, SD 8.8 and 48.5, SD 5.4 respectively; Supplementary Table 4).

Five participants had serious adverse events; four in the Concept group, with cow’s milk protein intolerance (3), and gastroesophageal reflux (1), diagnosed in Hospital or primary care. The first two received the intervention formula for 7 and 8 weeks respectively, whereas the other two for only 3 days, before being prescribed a different IF. One infant in the Control group was diagnosed with cow’s milk protein allergy and received the intervention formula for 9 weeks, before being prescribed a different formula. None of these diagnoses were considered to be related to the study product.

## Discussion

We report the growth and tolerance of LMPT infants from birth to 3 months corrected age comparing a concept IF containing large lipid droplets comprising a mixture of dairy and vegetable lipids and coated with milk phospholipids to a standard term IF (Control IF). This small-scale study could not confirm non-inferiority in daily weight gain up to 3 months corrected age for the concept group compared to the control group, whereas non-inferiority was demonstrated compared to the breastfed reference group and did not reveal any statistically significant differences in growth outcomes between the randomized groups, The larger gain in head circumference-for-age z-score and a larger increase in bone mineral content for the Concept group were not significant, and there were no other apparent differences in growth, body composition or gastrointestinal tolerance were observed. The small sample recruited in this trial and its exploratory nature, limit the interpretation of these findings.

Previous RCTs in healthy, term infants^25,26^, showed that a similar concept IF supported an equivalent daily weight gain for the first 4 months of age in comparison to either a standard formula or breastfeeding^25^. In line with these findings, the current study shows that the daily weight gain and other secondary growth outcomes of LMPT infants receiving the concept IF were not significantly different from infants receiving a standard term IF, or from breastfed infants. We have not been able to demonstrate non-inferiority in daily weight gain for the Concept compared to the Control group, but that was performed compared to the breastfed reference group. The current study suffered from lack of power due to the much smaller than intended sample size. Moreover, some apparent differences in baseline characteristics were observed between both randomized groups, of which the fact that the infants in the Concept group were randomized at almost 2 weeks earlier corrected age compared to the Control group, is likely to have affected the primary outcome. Our findings agree with those of Breij et al. ^25^ and Teoh et al. ^26^ with regards to gains in the z-scores of length-for-age and head circumference being similar for the Concept group compared to the Control group, showing adequate growth. In our LMPT cohort, infants in the Concept group also had larger increases in these parameters until 3 months corrected age, although the results are not significant, which may be due to the small participant numbers.

We noted that infants who were randomized to receive the concept IF had a bigger reduction in weight-for-age z-score prior to starting the intervention formula, between birth and randomization. Length-for-age also declined in the same period for this group, whereas head circumference-for-age did not seem to be affected. We cannot confidently explain this finding. We assume that the bigger reduction in weight-for-age for the Concept group prior to the intervention period may be related to the lower corrected gestational age at which these infants were randomized, which was closer to the physiological weight loss observed after birth. We performed a regression analysis of the weight-for-age on feeding group and gestation at randomization which supported this notion (data not presented). There were slightly more births via C-section in this group and more infants receiving antibiotics after birth, which theoretically could impact the microbiome and body weight. Also, these infants achieved full oral feeds at a later age, which may mean they received intravenous dextrose solutions in the first days of life (data not collected), potentially resulting in a bigger weight-for-age loss.

Studies have shown that the characteristics of the lipid droplets impact lipid metabolism in the short- and long-term^32,33^. Teller et al. ^34^, Oosting et al. ^35^ and Kodde et al. ^36^ have suggested in experimental studies that early diet with larger lipid droplets coated with milk phospholipids had improved body composition and metabolic biochemical markers in adult life of rats compared to those fed with standard IF in early life. In this study, we found no apparent differences in the body composition until 3 months corrected age.

Longer-term outcomes of the current concept IF have not been reported previously in late or moderate preterm infants. In a follow-up study of a 4-month IF intervention trial, term-born children fed the concept formula had lower BMI compared to the control formula, closer to that of breastfed reference infants, and lower blood pressure at 5 years compared to children fed the control formula in early life^37^. Moreover, compared to control formula, the infants who received the concept formula demonstrated some positive neurocognitive outcomes at 5 years of age^38^. Timby et al. found that infants fed until 6 months of age with an IF supplemented with milk fat globule membrane, had better developmental score at 1 year compared to those fed a standard formula^39^. We found a non-significant larger increase in the head circumference of infants fed the concept IF compared to the control IF, which could be associated with better neurodevelopmental outcomes in later life ^40,41^.

Our study design was in conjunction with the Baby Friendly Initiative. Parents were approached to take part in the RCT only after they, independently, had started to exclusively formula feed their infants and they had no knowledge of the RCT prior to this. This protected the process of establishing breastfeeding, which is more complex in LMPT infants^42^. Families who joined the cohort made their own decisions about duration and exclusivity of breastfeeding.

However, there were certain limitations to our study. The final number of participants was significantly smaller than intended due to a low consent rate of formula feeding infants and, the COVID pandemic which prevented face-to-face follow-up and resulted in early termination of recruitment. Also, there were attrition losses early in the study, before the cut-off age of 4 weeks corrected age, which did not allow us to discuss with the parents and recruit more infants in the RCT. To improve recruitment, we adapted the inclusion criteria by allowing a higher birth weight (up to 3 kg) and older age at randomization (up to 4 weeks after TEA). As a consequence, 6 infants had a birth weight >2.5 kg (4 in control group and 2 in the Concept group), although the mean birth weight remained well below 2.5 kg for both intervention groups (Table 1). Thirteen infants were randomized in the RCT after TEA (8 in the control group and 5 in the Concept group), of which only 4 were randomized a week or more after TEA and the mean corrected gestation of randomization remained <40 weeks for both groups (Table 1). Therefore, we believe that the amendment of the inclusion criteria had limited impact on the overall cohort characteristics. The small number of participants did not allow us to perform the originally planned statistical analysis^27^ or to stratify the results as planned or perform an adjusted regression analysis, for example taking sex or gestational age into account. We also noted a low rate of families completing the 7-day-diary (49% families in total, Supplementary Table 4). Finally, the two-step approach and inclusion in the RCT, meant that infants were randomized close to TEA. Although the type of feeding milk prior to this can be a confounding factor as it may impact some growth and body composition, preserving the Baby Friendly Initiative was a priority.

In conclusion, our findings suggest that the concept formula supports adequate weight gain in LMPT infants and was well tolerated. Our findings should be taken with caution, in view of the small population size, and high-quality, adequately powered RCTs are required. However, they suggest that the concept IF could be a safe alternative for LMPT infants whose parents have decided to formula feed.

## Supplementary information

Supplementary information

Supplementary Tables

Supplementary information

## Data Availability

The datasets generated during and/or analyzed during the current study are available from the corresponding author on reasonable request.
