# Supplementary material for: Formula with large, milk phospholipid-coated lipid droplets in late-moderate preterm infants: a double-blind RCT
Source: Pediatr Res. 2024 Sep 18;97(2):639–46. doi: 10.1038/s41390-024-03476-x (PMC12014481; doi:10.1038/s41390-024-03476-x)
Supplement: Supplementary file 2 — Supplementary Tables [file 41390_2024_3476_MOESM2_ESM.pdf]

SUPPLEMENTARY TABLES

Supplementary Table 1. Composition of the intervention products (per 100 ml)

Per 100 ml	Control IF	Concept IF
**Energy (kcal)**	66	66
**Fat (g)**	3.4	3.5
**Vegetable oil (g)**	3.3	1.6
**Milk Lipids (g)**	0.1	1.9
**Saturates**	1.5	1.7
**Monounsaturates**	1.3	1.2
**Polyunsaturates**	0.6	0.6
**Linoleic acid (mg)**	445	422
**Alpha linolenic acid (mg)**	82	78
**Arachidonic acid (mg)**	11	12
**Eicosapentaenoic acid (mg)**	2.2	2.5
**Docosahexaenoic acid (mg)**	10	11
**Palmitic acid (mg)**	581	633
**sn-2 Palmitic acid (mg)**	58	222
**Milk Phospholipids (mg)**	-	56
**Soy Phospholipids (mg)**	4.2	-
**Protein (g)**	1.3	1.3
**Whey protein (g)**	0.8	0.8
**Casein (g)**	0.5	0.5
**Carbohydrates (g)**	7.3	7.1
**scGOS/lcFOS (9:1)(g)**	0.8	0.8
**Vitamins**		
**Vitamin A (µg RE)**	54	59
**Vitamin E (mg α-TE)**	1.1	1.0
**Alpha-tocopherol (mg)**	1.3	1.1

IF, infant milk formula; RE, retinol equivalent; scGOS/lcFOS (9:1), a specific prebiotic mixture consisting of short-chain galactooligosaccharides and long-chain fructo-oligosaccharides in a ratio of 9:1; α-TE, α-tocopherol equivalent.

Supplementary Table 2. Demographics of the reference group and the full study cohort

	**Breastfeeding**	**Full cohort (following attrition losses)**
**n**	39	107
**Male**	19 (49%)	52 (49)
**Female**	20 (51%)	55 (51%)
**Gestation (mean)**	34.99 (1.26)	35.3 (34.1, 36.3)
**MPT**	9 (23%)	24 (22%)
**LPT**	30 (77%)	83 (78%)
**Mode of delivery**		
**Normal vaginal delivery**	16 (41%)	53 (49.5%)
**Elective C-section**	7 (18%)	22 (20.6%)
**Emergency C-section**	16 (41%)	32 (29.9%)
**Singleton**	33 (85%)	77 (72%)
**Multiple**	6 (15%)	20 (18%)
**Maternal age (mean)**	33 (4.5)	33 (29, 36)
**Maternal BMI (median)**	24.2 (22-27.1)	24.6 (22.0, 30.1)
**Admitted to NNU**	17 (43.6%)	52 (48.6%)
**Postnatal antibiotics**	19 (43.6%)	57 (53.3%)
**Corrected gestation at study entry (median)**	36.4 (35.3-37.1)	

MPT: moderate preterm, LPT: late preterm, BMI: body mass index,

Supplementary Table 4. Feeding intake, behaviour and gastroesophageal tolerance and symptoms at 3 months corrected age

	Control	Concept	p-value
**IGSQ score [b]**	20 (20, 28)<br>n=15	22 (18, 25)<br>n=15	0.74
**Average stool frequency [a]**	1.15 (0.84)<br>n=11	1.19 (0.78)<br>n=9	0.93
**% days with watery stools [b]**	14.3 (0, 25)<br>n=11	0 (0, 43)<br>n=9	0.97
**% days with hard stool(s) [b]**	0 (0,0)<br>n=11	0 (0, 0)<br>n=9	0.73
**Average stool type (1=watery, 2=soft, 3=formed, 4=hard) [b]**	1.88 (1.67, 2.0)<br>n=11	1.71 (1.2, 2.0)<br>n=9	0.30
**% days with cramps [b]**	0 (0, 13)<br>n=11	0 (0, 0)<br>n=9	0.55
**% days with regurgitation [a]**	58% (45.5)<br>n=11	18.1% (34.7)<br>n=9	0.05
**% days with vomiting [b]**	13 (0, 100)<br>n=11	11 (0, 29)<br>n=9	0.47
**% days with nappy rash [b]**	13 (0, 25)<br>n=11	0 (0, 0)<br>n=9	0.16
**Formula intake (ml/kg/d) [a]**	138.2 (22.5)<br>n=11	153.7 (22.3)<br>n=9	0.14
**BEBQ score [a]**	48.5 (5.4)<br>n=17	47.9 (7.0)<br>n=15	0.77
**Iron supplements [n (%)] [c]**	9 (53%)	7 (57%)	0.67

[a] Results are presented as mean with standard deviation and p-value calculated using t-test

[b] Results are presented as median with quartile 1, quartile 3 and p-value calculated using Mann-Whitney test

[c] p-value is calculated using chi-square test

IGSQ= infant gastrointestinal symptoms questionnaire, BEBQ= bottle emptying behaviour questionnaire

When IGSQ or BEBQ were incomplete, the total score was not calculated and that infant was not included in the statistical analysis
